# In-Depth Characterization of PEGylated Liposomes: Using AF4 and HPLC-CAD in Tandem as a Strategy for Composition Assessment and Postinsertion Optimization

**DOI:** 10.1021/acs.analchem.5c07710

**Published:** 2026-06-25

**Authors:** Silvia L. Appleton, Guillaume Bucher, Jessica Ponti, Dora Mehn

**Affiliations:** 99013 European Commission, Joint Research Centre (JRC), Ispra 21027, Italy

## Abstract

The surface of nanoparticles for pharmaceutical use is crucial as it is the first interface that interacts with the biological environment in the body and therefore determines the fate of the nanoparticle itself. Aware of its key role, regulatory agencies urgently require rigorous quality control of the surface, including polyethylene glycol (PEG) coatings, a component widely used in nanomedicine, which is also present in recent vaccines against SARS-CoV-2, to ensure efficacy and safety. Building on a review of the literature and experimental testing, this study proposes a workflow to overcome critical issues related to liposome PEG coating. It integrates microfluidic synthesis with PEG postinsertion, using mild incubation conditions and a straightforward quality control analysis for method efficiency and reliability. Using multidetector Asymmetric-Flow Field-Flow Fractionation (AF4-MD) in combination with High-Performance Liquid Chromatography coupled to a Charged Aerosol Detector (HPLC-CAD) proved to be an efficient strategy for analyzing simultaneously both the size distribution and other size-dependent parameters with special attention on the precise composition of PEGylated liposomes. By exploiting the AF4 size separation of PEGylated liposomes from unincorporated PEG-lipid micelles, this approach enables the optimization of postinsertion reaction conditions and the validation of traditional purification methods. This comprehensive workflow allows for in-depth liposome characterization, focusing on critical quality attributes. It includes reliable and straightforward PEG coating determination, essential for postinsertion tuning, and offers potential adaptability to other stealth coatings, thus providing a valuable reference for nanomedicine developers, control laboratories, and regulatory bodies.

## Graphical abstract

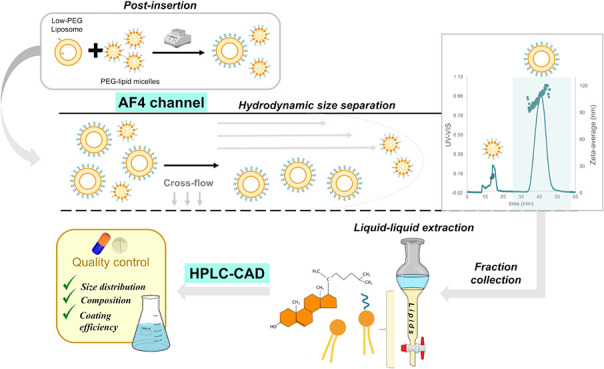

PEGylation, the coating of lipid membranes with poly­(ethylene glycol) (PEG) polymers, is a well-known strategy for lipid nanoparticle formulation. Their protein-repellent property makes nanoparticles less visible to the immune system, extending their half-life and, consequently, improving their therapeutic efficacy. This crucial feature has given rise to the “stealth” class of nanoparticles, a term that reflects their immune-evasive capabilities, particularly exemplified by stealth liposomes, for which this term was originally coined.^1,2^ Doxil, Caelyx, Onivyde, and Lipodox^3^ are clinically significant examples belonging to this class. It is important to note that this strategy has also been adopted for solid lipid nanoparticles as life-saving vaccines against SARS-CoV-2.^4,5^ Therefore, given the importance of these nanocarriers, whose surfaces have become increasingly sophisticated due to the presence of coating polymers and ligands for active targeting, regulatory authorities^6,7^ have emphasized the urgent need to assess their critical quality attributes (CQA). These attributes include overall composition, internal structure, and loading,^8,9^ as well as their external surface,^10–13^ as outlined by the European Medicines Agency (EMA) in the reflection paper on coated nanomedicine products,^14^ which is responsible for interaction with biological fluids and determines internalization into target cells. Importantly, even small differences on the surface are sufficient to cause significant variations in the biological response and may therefore have a major impact on efficacy and safety.^10,15–17^ Recent studies have revealed that PEG can in fact be recognized by the immune system in a number of patients, triggering the production of anti-PEG antibodies.^18^ These antibodies can attach to PEG-coated pharmaceuticals, accelerating their clearance and reducing their effectiveness, and in some cases resulting in allergy-like reactions. This has stimulated further research into the relationship between PEG chemistry and the immune system,^19^ emphasizing the importance of a comprehensive characterization of PEGylated nanocarriers to enhance formulation design and minimize potential adverse effects.

As far as liposome research is concerned, below we briefly outline the context, starting from the PEG coating methods and then moving on to analytical characterization, identifying the critical points that can lead to poor-quality or insufficiently characterized formulations and that inspired the approach proposed in this work.

Alongside postsynthetic surface modification, which is extensively discussed elsewhere,^20^ PEG coating is usually achieved using two methods, namely pre- and postinsertion, in which PEGylated lipids are added before or after liposome assembly, respectively.^12^

One major advantage of postinsertion is that it allows for a more controlled PEG coating. This is because, unlike in preinsertion, the PEG chains do not orient themselves randomly toward the inside and outside of the liposome’s internal cavity, but only toward the outside.^12^ This uniform outward orientation considerably simplifies the characterization of the surface composition of liposomes, making quality control more robust, which is the main objective of this study.

Several aspects must be considered when developing the postinsertion method to ensure that optimal formulations are achieved. These include the synthesis of PEG-free liposomes and PEG-lipid micelles, the selection of incubation temperature and time, the choice of organic solvents, and the purification strategy. In fact, excessively laborious, multistep synthesis processes^21^ requiring high temperatures or long incubation time may cause degradation of lipid components, loaded drugs, and ligands for targeting conjugated on the surface. In addition, traditional purification methods may fail to completely remove unincorporated PEG-lipids, which could interfere with the accurate and reliable characterization.

As demonstrated in this study, careful optimization of postinsertion and purification assessment is crucial for ensuring consistent and reproducible results, which we have achieved using a size-separation technique, that is, asymmetric-flow field-flow fractionation (AF4) for precise evaluation of PEG coating efficiency.

AF4 is a flow field-based fractionation technique that can be coupled in-line to multiple detectors (AF4-MD), including refractive index, UV–visible absorption, fluorescence, circular dichroism, dynamic light scattering (DLS), multiangle light scattering, and recently, nanoparticle tracking analysis (NTA)^16^ and small-angle X-ray scattering,^9,22^ allowing for an exceptionally thorough characterization that overcomes the limitations of batch-mode DLS, which is less reliable in analyzing polydisperse samples, as seen in nanoparticulate pharmaceutical systems. Unlike chromatographic techniques, such as size exclusion chromatography, AF4-MD does not have a conventional stationary phase that could alter the nanoparticles due to interacting shear forces.^23^ It gently separates the nanoparticles that elute in a parabolic laminar flow profile of the liquid mobile phase in a thin channel, making it suitable for organic nanoparticles such as liposomes and other lipid-based nanoparticles, extracellular vesicles, and polymeric particles.^23^ The ability to conduct characterization directly in the nanoparticle dispersing medium, e.g., saline solution or cell culture media, makes AF4 particularly promising. This capability translates effectively into the pharmaceutical sector, where it aids the prediction of both efficacy and safety.^24^ As a result, there is growing literature highlighting the role of AF4 in drug development regarding manufacturing monitoring and final drug product validation,^25,26^ as well as in the analysis of the protein corona after exposure to biological fluids.^27^ The latter enables precise predictions of the biological fate of the nanomedicine, thus providing opportunities to optimize the formulation and demonstrating the versatility of AF4.

Its role in the characterization of nanomedicines has also been recognized from a regulatory perspective, enhancing its position in terms of prospects and reliability. It has been central to the Standard Operating Procedures (SOPs) developed by the US National Cancer Institute Nanotechnology Characterization Laboratory (NCI-NCL),^28^ the European Union Nanomedicine Characterization Laboratory (EUNCL),^29^ regulatory bodies (EMA and FDA), and the NIST metrology institute, aiming to support the pharmaceutical community in addressing the essential regulatory requirements for liposome preclinical characterization.^23^ Furthermore, the newly updated ISO standard entitled “Nanotechnologies—Analysis of nano-objects using asymmetrical flow and centrifugal field-flow fractionation”^30^ underscores the current significance and applicability of this technique.

In this study, we developed and tested an analytical protocol for nanomedicine quality control using liposomes synthesized in-house. Our formulation method combined microfluidics with postinsertion, employing mild incubation conditions to explore various doses of PEG-lipid and PEG chain lengths, specifically PEG2000 and PEG5000.

Using AF4-MD, we successfully separated unincorporated PEG-lipid micelles from PEGylated liposomes through gentle size-based separation within the same aqueous environment (PBS) as for the liposomes. This approach facilitated reliable assessment and optimization of postinsertion efficiency by integrating signals from UV and DLS in-line detectors, enabling real-time monitoring of micelle-liposome separation while simultaneously analyzing liposome size distribution and integrity.

To obtain precise composition information about the PEG coating, we developed a rapid, efficient, and reproducible liquid–liquid lipid extraction method. This allowed AF4 fractions containing PEGylated liposomes free from unincorporated micelles to be analyzed through off-line HPLC-CAD, using a method adapted from ASTM standards. Additionally, our workflow confirmed the stable coating of PEG chains on liposomes in cell culture medium and validated commonly used laboratory purification methods essential for the formulation process.

## Materials

### Lipids

All lipids were purchased from Merck/Avanti Polar: 1-palmitoyl-2-oleoyl-glycero-3-phosphocholine (POPC, Cat. #850457C); Cholesterol (Cat. #C8667); 1,2-distearoyl-*sn*-glycero-3-phosphoethanolamine-*N*-[methoxy­(polyethylene glycol)-2000] (18:0 PEG2000 DSPE, Cat. #880120P), 1,2-distearoyl-*sn*-glycero-3-phosphoethanolamine-*N*-[methoxy­(polyethylene glycol)-5000] (ammonium salt) Cat. #A88220P.

### Buffers

The buffers were provided by Thermo Fisher Scientific: Phosphate-buffered saline (PBS, Cat. 70011044) was used for liposome synthesis and dialysis, whereas Gibco PBS Tablets (Cat. 11510546) were used for AF4 mobile phase preparation.

## Methods

### Liposome Synthesis

Liposome batches were synthesized using a microfluidic device (NanoAssembler Ignite, Cytiva). POPC, cholesterol, and DSPE-PEG2000 were dissolved in pure ethanol at a molar ratios of 52:45:3, 54.8:45:0.2, and 55:45:0 for PEG 3 mol %, 0.2 mol %, and 0 mol %, respectively, to reach a total lipid concentration of 10 mg/mL. The lipid ethanol solution and 1X PBS at pH 7.4 were mixed through a commercial microfluidic cartridge (NanoAssemblr Ignite NxGen cartridges, Cytiva) at a flow rate ratio of 1.5:1 (lipid:aqueous) and a total flow rate of 12 mL/min.

The formulations were dialyzed in PBS pH 7.4 for 24 h at room temperature using dialysis cassettes with a molecular weight cutoff (MWCO) of 3,500 (Pur-A-Lyzer dialysis kit, Sigma-Aldrich, Cat. #PURD35050).

### Postinsertion

DSPE-PEG2000 and DSPE-PEG5000 micelles were prepared by dissolving the lipids in PBS at a concentration of 14.17 and 36.74 mg/mL, respectively, for 15 min at 40 °C. They were then characterized using DLS in batch mode without dilution to verify their size and polydispersity index. Once the successful formation of micelles was confirmed, an aliquot corresponding to 0.75, 1.5, 3, 6, and 10 mol % was added to the PEG 0.2 mol % liposome batch and incubated under gentle agitation at 37 °C for 1 to 5/7 h depending on the objective of the experiment. At the end, each reaction mixture was washed three times with PBS using centrifugal filter devices (AMICON, cutoff 100 kDa, Millipore) and centrifuged at 2000 × *g* and then characterized for quality control, leaving an aliquot for AF4 injection to evaluate coating efficiency.

Expected maximal chain length difference between PEG2000 and PEG5000 could be about 24 nm if the chains were fully extended—based on the length of a monomer. On the other hand, at the applied PEG-lipid concentration the chains are not expected to appear as a fully extended brush on the surface and the expected thickness of the PEG layer is supposed to fall in the 3.7–4.2 nm range.^31^

### Asymmetric-Flow Field-Flow Fractionation

Liposomes diluted 1:1 in PBS pH 7.4 were injected in AF2000 Multiflow FFF (Postnova Analytics) equipped with an isocratic pump, degasser, injector, and a fractionation channel. UV–vis absorbance and DLS were selected as the in-line detectors. The AF4 fractions, once collected, were concentrated with centrifugal filter devices (AMICON, cutoff 100 kDa, Millipore) up to 1 mL and used for lipid liquid–liquid extraction for subsequent HPLC-CAD analysis. The measurement conditions for MD-AF4 analysis are reported in Table S9.

To check the feasibility of the AF4-MD method, sample recovery was determined by comparing the UV area under the curve of liposome PEG 0.2 mol % analyzed with an in-line UV–vis detector at a wavelength of 280 nm in the presence and absence of the crossflow, the coefficient of variation (COV) of the repeatability for the peak retention time (UV–vis detector) and size (online DLS).

### Dynamic Light Scattering

DLS measurements were conducted using a Zetasizer Nano ZS (Malvern Instruments, Worcestershire, UK).

Flow mode: the backscattering detector (173°) was used for measuring the hydrodynamic diameter; the measurements were made in a quartz flow cell (Malvern ZEN0023) and data was collected using Malvern Zetasizer software (v7.11). The intensity threshold was manually adjusted after each experiment.

Batch mode: the liposomes were diluted 10-fold with PBS pH = 7.4 and analyzed in semimicro PMMA cuvettes (BRAND UV cuvette micro, H 8.5 mm, 70–850 μL, Sigma-Aldrich). The zeta average size (nm) and PDI of liposomes and micelles were measured at 25 °C at a 173° backscatter angle, using the parameters of viscosity 0.8872 cP, refractive index 1.33, and equilibration time of 180 s. Three consecutive acquisitions, each one average of 10 individual measurements, were conducted for each sample.

### Electrophoretic Light Scattering

ELS measurements were performed on liposome batches diluted 1:20 in ultrapure water at pH 7.4 using a Zetasizer Nano ZS (Malvern Instruments, Worcestershire, UK) at room temperature. The cuvettes DTS1070 (Malvern) were used for the analyses.

### Liquid–Liquid Extraction of Lipids

The liposome PEG 3 mol % was diluted 1:100, 1:200, 1:400, and 1:1000 in 1 mL of PBS, and to each dilution 200 μL of chloroform was added to perform a liquid–liquid extraction. The mixtures were subjected to shaking and vortexing for 1 min. Then they were centrifuged at 1000 rcf for 1 min at room temperature to facilitate organic-aqueous phase separation. The lower organic phase, containing the extracted lipids, was collected using a pipet and transferred in empty Eppendorf tubes. The upper aqueous phase was extracted a second time following the same procedure, and the organic phase was added to that of the first extraction and left to evaporate at room temperature overnight, while the remaining aqueous phase was discarded. Once dry, the lipids were dissolved in 200 μL of methanol, vortexed for 1 min, and sonicated for 1 min. These lipids were subsequently ready for HPLC-CAD injection for composition analysis.

### High-Performance Liquid Chromatography

Liposome lipid components quantification was adapted from the ASTM standard method E3297–21, using a Thermo-Dionex 3000 RS HPLC system fitted with a Waters xBridge RP HPLC BEH C18 column (13 nm pore size, 3.5 μm particle size, 3 mm × 150 mm) and coupled to a Charged Aerosol Detector (CAD), Thermo-Dionex Corona Veo RS. Details on the optimized gradient elution for lipid separation are reported in Figure S2. Calibration was performed in the range of 2.5 to 300 μg/mL for all lipids. The practical LOQ was defined by the first calibration standard (i.e., 2.5 μg/mL) for all tested lipids. The accuracy on the determination of all tested lipids in synthetic QC samples (a mixture of lipids in methanol) was in the range of 85% to 115% at the LOQ level and 95% to 105% above the LOQ level. The relative standard deviation (RSD) of lipid content assessed in synthetic QC samples (a mixture of lipids in methanol) was on average <5%, thus demonstrating good repeatability and precision.

### Validation of Dialysis and Ultrafiltration Washing

Dialysis: Liposome PEG 0.2 mol % was synthesized and dialyzed for 24 h in PBS. Then it was diluted 1:50 in methanol, vortexed for 1 min, and analyzed with HPLC-CAD. An aliquot of this batch was then injected in AF4 and the fraction containing the liposomes was collected, extracted, and analyzed with HPLC-CAD. The liposome composition was therefore put into comparison after dialysis and AF4.

Centrifugal ultrafiltration washing: Liposome PEG 0.2 mol % was incubated with 3 mol % DSPE-PEG2000 at 37 °C for 1 and 5 h to simulate unsuccessful and successful postinsertions. From each reaction mix, 20 μL was injected in AF4 and the fractions containing liposomes were collected and used for liquid–liquid extraction of lipids for HPLC-CAD analysis.

The remaining batches were washed 3 times with PBS using centrifugal filter devices (AMICON, regenerated cellulose, cutoff 100 kDa, Millipore) and centrifuged at 2000 rcf speed; they were then ready for 1:100 dilution in methanol for HPLC-CAD analysis. The liposome composition was therefore put into comparison after centrifugal ultrafiltration and AF4.

### Stability

Liposomes postinserted with 3 mol % were mixed in a 1:1 ratio with sterile phenol red-free DMEM cell culture medium containing 10% FBS and incubated overnight at 37 °C under mild agitation. A volume of 20 μL was injected into AF4 and monitored by UV detection at 280 nm and DLS connected in-line. The AF4 fraction containing liposomes complexed with serum proteins was collected and concentrated to 1 mL final volume with centrifugal filter devices (AMICON, regenerated cellulose cutoff 100 kDa, Millipore). The AF4 fractions were then used for the liquid–liquid extraction of lipids and analyzed with HPLC-CAD.

### Cryo-TEM Analysis

Transmission electron microscope (TEM; JEOL JEM-2100, JEOL, Italy) was used at 120 kV for cryo-TEM analysis to characterize the morphology of liposomes postinserted with 3 mol % PEG-lipid. An aliquot of 4 μL, in 10% sucrose, was cryo-fixed on a Formvar holey carbon film grid, pretreated by glow discharge (10 mV, 30 s, Leica EM ACE 200, Microcontrol, Italy) by plunge freezing technique (Leica EM GP, Microcontrol, Italy). Three hundred particles were selected to calculate size distribution by Image-J Fiji V5. Results are reported as Feret min (mean ± standard deviation).

## Results and Discussion

### Synthesis of Liposomes with Low-PEG Content via Microfluidics

The synthesis of liposomes using microfluidics has become very popular due to its ease of use and reproducibility. It consists of the controlled mixing of lipid components with an aqueous buffer under laminar flow in microfluidic channels, resulting in the spontaneous formation of unilamellar liposomes. Furthermore, computer-controlled injections allow the size of the liposomes to be finely tuned.^32^

The current postinsertion methods involve the synthesis of PEG-free liposomes through traditional thin-film hydration, followed by extrusion for nanoparticle size selection and incubation with PEG micelles produced via the same route.^3^

Thin-film hydration is, however, a multistep process that requires specific equipment for extrusion, rotary evaporation, and ultrasonication for the rehydration of lipid films. As described in detail in the literature,^3^ many precautions must be taken for each process, as problems related to formulation stability may arise due to heating, possible sample loss during evaporation, failure of liposomes to exit the extruder, and cross-contamination, making the entire process laborious.

Our objective was therefore to synthesize stable liposomes with low PEG content using microfluidics as a platform for subsequent postinsertion, offering an alternative to thin-film hydration.

After determining experimentally that PEG-free liposomes synthesized through microfluidics without an extrusion step were unstable and therefore unsuitable, we adjusted the synthesis protocol. We included a minimal amount of PEG-lipid to ensure formulation stability and minimize the random orientation of PEG chains toward the interior of the liposome, which negatively impacts the accuracy of liposome surface characterization.

For this purpose, a series of liposomes was prepared by keeping the ratio of cholesterol to total lipids constant while varying the proportions of the other two lipid components, POPC and DSPE-PEG2000. Due to its critical role in determining membrane structure and fluidity, the cholesterol ratio was kept unchanged.^33^ More precisely, we reduced the preinserted DSPE-PEG2000 content from 3 mol % down to 0.2 and 0 mol %, compensating for this reduction by increasing the POPC content to maintain the total lipid amount.

The hydrodynamic diameter and PDI remained unchanged despite the reduction in PEG-lipid content down to as little as 0.2 mol % (Figure 1A and Table S1) and this is fully in-line with the expected, negligible change in the total diameter due to the PEG layer thickness in the actual, mushroom-like PEG conformation regime. PEG 3 mol % was used as a reference since it is a coating density commonly used in the literature to confer stealth properties to liposomes. In contrast, in the PEG-free formulation, batch-mode DLS showed a slight widening of the peak centered at a similar size, suggesting greater polydispersity. The heterogeneity of the sample was particularly evident in the AF4-DLS fractogram (Figure 1B), where it was possible to identify the population of nanoparticles eluting at 64 min responsible for the increase in size and PDI seen with DLS in batch mode. In addition, the zeta potential being close to zero (Table S1) suggested an increased tendency for particles to aggregate, which was validated by the appearance of a precipitate after 1 day. This highlights the significant role of PEG as a stabilizer during the formulation phase.

**Figure 1:**
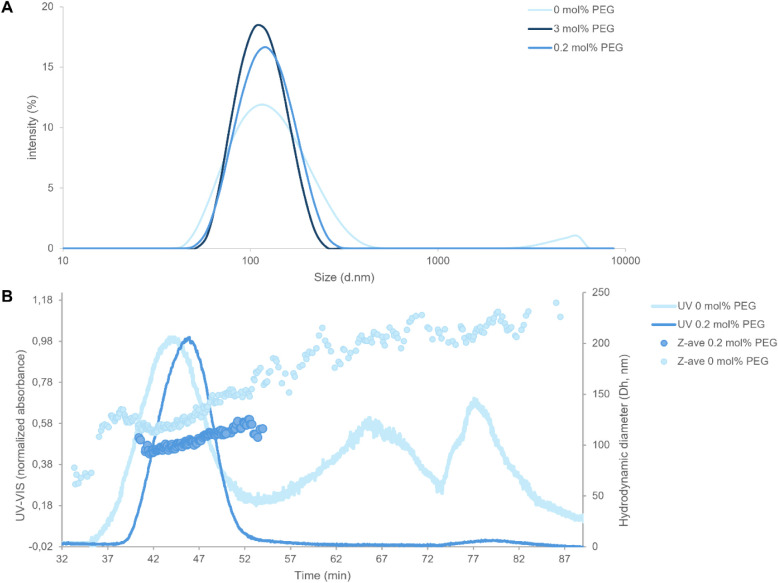
Low-PEG-content liposome microfluidic synthesis; A) Intensity-based particle size distribution by batch mode DLS comparing 0 and 0.2 mol % PEG with 3 mol % PEG; B) AF4-DLS fractogram of liposome batch 0 mol % PEG overlaid on 0.2 mol % PEG with the line plot representing the UV fractogram, whereas the scatter plot represents the Z-average of light-scattering colloids.

Given the repeatability of the synthesis across three independent batches (Table S1), we chose the PEG 0.2 mol % formulation as a platform for the subsequent postinsertion step to minimize the variability of the postinsertion process.

### Development of AF4-MD Method

We assessed the feasibility of the AF4-MD method with integrated UV signal-based recovery values greater than 70%, a relative standard deviation (RSD) of the repeatability for the peak retention time (UV–vis detector), and size (in-line DLS) below 0.5% (Table S2).

We also checked the correspondence of the hydrodynamic diameter determined by AF4-DLS with batch-mode DLS and it remained unchanged (Table S3), so the method did not destabilize the formulation but gently separated the liposomes based on their size.

To validate the efficiency of the AF4-MD method in separating unincorporated PEG-lipid micelles from liposomes, the mixture was injected into AF4. Both the UV signal and the in-line DLS light scattering intensity (kilocounts per second, kcps) showed two distinct peaks, namely, at 18.5 min for the micelles and at 44 min for the liposomes (Figure 2). In addition, the fraction corresponding to free PEG-lipid was collected and extracted through the liquid–liquid extraction method herein developed, and its identity was confirmed by HPLC-CAD (Figure S1).

**Figure 2:**
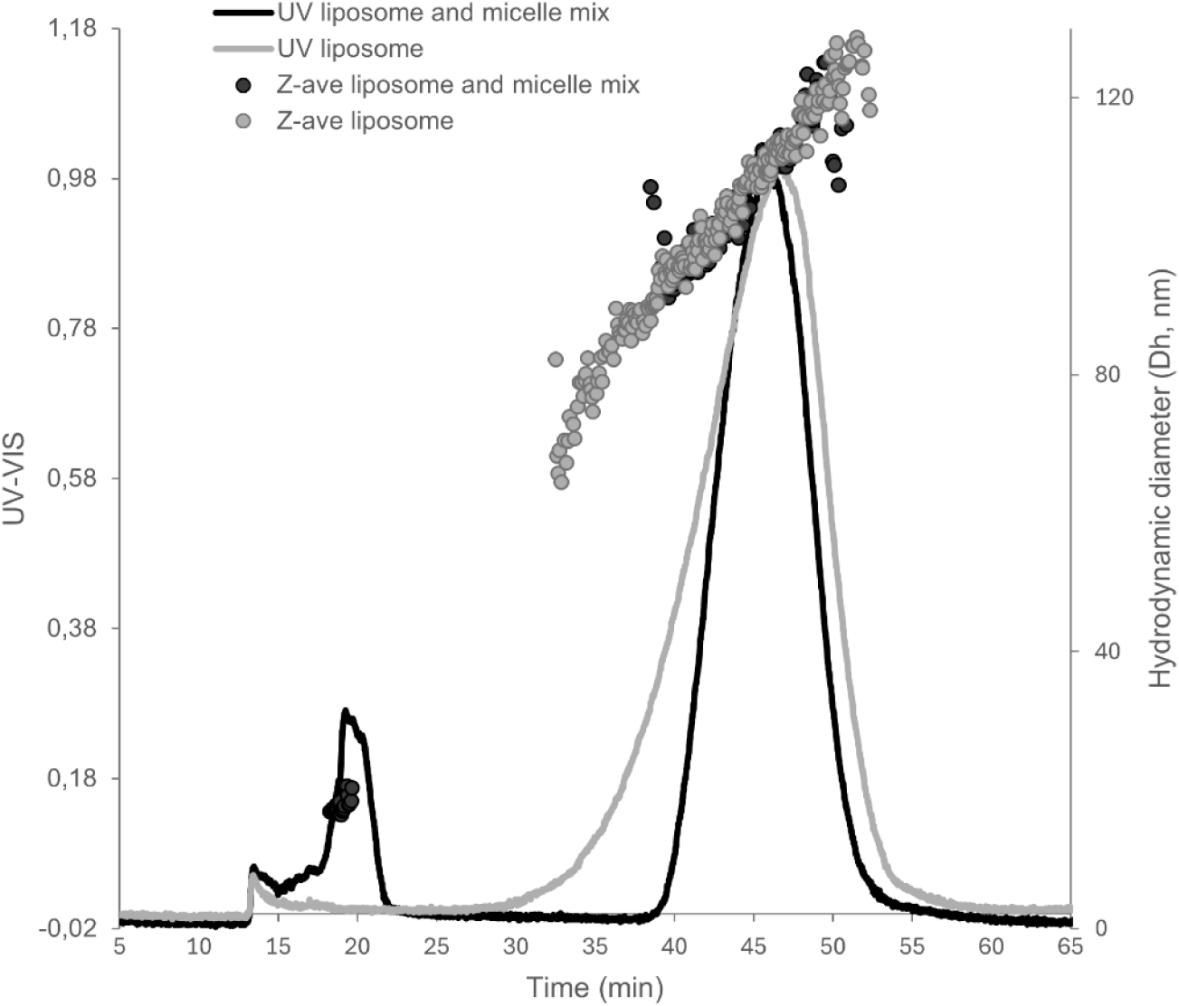
AF4-UV-DLS fractogram of liposome batch 0.2 mol % PEG, with and without PEG-lipid micelle spiking, measured at 280 nm (UV) and with the in-line DLS. The line plot represents the UV fractogram, whereas the scatter plot the Z-average of light-scattering colloids. The void peak appears at about 14 min.

We therefore adopted this method as the analytical strategy to characterize postinserted liposomes free from unincorporated PEG-lipids and evaluate PEG coating efficiency.

### Optimization of Lipid Extraction from AF4 Fractions and HPLC-CAD Analysis

The objective was to develop an analytical method to determine the composition of liposomes purified using AF4, keeping in mind that the postdetector fraction contained highly diluted liposomes (more than 500 times compared to the injected concentration) in PBS with the risk of falling below the limit of quantification. Importantly, the salts contained in PBS may not be compatible with all detection systems, such as mass spectrometry and CAD,^34^ as they may clog the nebulizers, increase background noise and thus reduce sensitivity.

The ASTM standard E3297-21 ^35^ describes a validated HPLC-CAD method for the quantitation of lipids in liposome formulations. It allows lipids to be analyzed without the use of fluorescent probes, which are generally employed for detection rather than therapeutic purposes, thereby making quality control less straightforward. Interestingly, simultaneous quantification of lipid and nucleic components is possible^36^ and has recently been used for impurity profiling of the stealth PEGylated lipid excipient in the Spikevax mRNA vaccine,^37^ demonstrating the technique’s current relevance and versatility. For these reasons, we selected HPLC-CAD for this study using a method adapted from the aforementioned ASTM standard to ensure complete separation of the chromatographic peaks of the lipid components, thereby meeting the resolution requirements specified in the European Pharmacopoeia (Figure S2).

It was also preferred to the spectrophotometric iodide assay for PEG detection,^3^ as we needed an analytical method capable of simultaneously quantifying all lipid components alongside DSPE-PEG (Figure S3), to check also the integrity of the liposomes after postinsertion and elution in AF4.

Due to the incompatibility of the presence of PBS with CAD, the traditional liquid–liquid extraction method for lipids, called the Bligh and Dyer method,^38^ was tested on 3 mol % PEG liposomes in PBS to cover a wide range of dilutions (Table S4). Lipid recovery at dilution factors of 200 and 80 ranged between 40% and 60%, with DSPE-PEG showing particularly low recovery (Figure S4), the Bligh and Dyer extraction method was therefore unsuitable, and consequently an alternative protocol was developed and optimized.

The alternative extraction protocol consisted of a rapid double liquid–liquid extraction using chloroform and was tested using 3 mol % PEG liposomes with dilution ranging from 1:100 to 1:1000 in PBS to mimic the concentrations of liposomes typically found in AF4 fractions (Figure 3).

**Figure 3:**
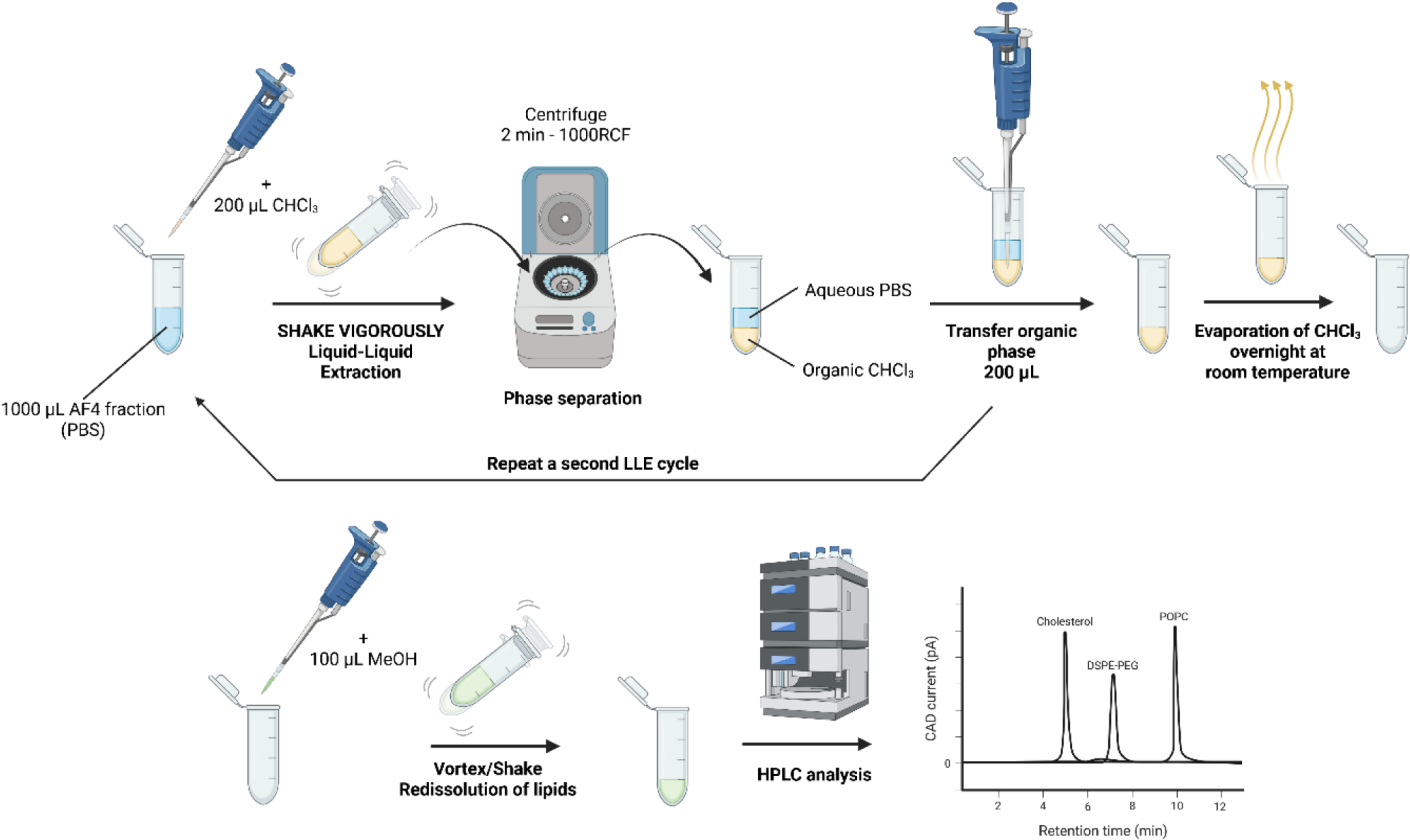
Schematic representation of the liquid–liquid extraction of lipids workflow. Created in BioRender. Bucher, G. (2026) https://BioRender.com/m0cm3pe.

The absolute recovery was over 79% for all lipids across these dilutions (Table S5). The relative recovery, normalized to cholesterol content, was 98% and 89% for DSPE-PEG2000 and POPC, respectively (Table S6 and Figure 4).

**Figure 4:**
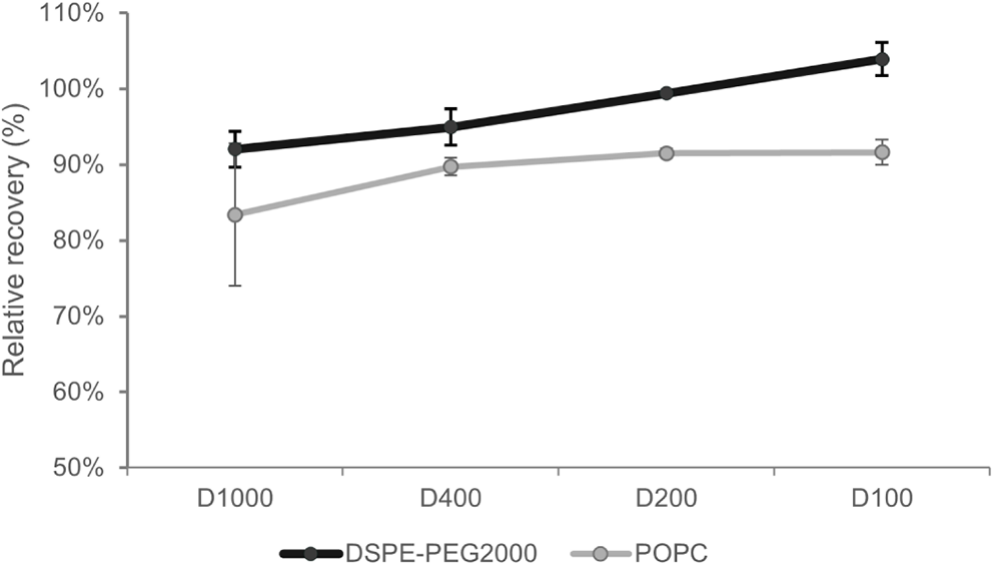
Relative recovery normalized to the cholesterol content after liquid–liquid extraction of DSPE-PEG2000 and POPC.

To conclude, salts were successfully removed, and lipids were concentrated in AF4-simulating fractions, enabling quantification through CAD, as demonstrated by the consistent extraction efficiency even at high dilutions. In addition, having covered a wide range of dilutions in AF4-simulated fractions allowed us to understand the minimum concentration of lipids that can be extracted in a repeatable manner. We consequently optimized the preparation of real samples, i.e., liposomes collected after running in AF4, before proceeding with extraction for HPLC-CAD analysis. In fact, considering the greater variability in POPC extraction efficiency at a dilution of 1:1000, the real samples were concentrated through centrifugal ultrafiltration in order to have an initial lipid quantity that falls within the other three dilutions.

This extraction method has made it possible to combine the AF4 size separation with nanoparticle composition analysis using HPLC-CAD, thus opening the possibility of both verifying and optimizing postinsertion and validating purification techniques commonly used at laboratory scale, such as dialysis and centrifugal ultrafiltration.

### Postinsertion Tuning

Postinsertion requires the preparation of PEG-lipid micelles, commonly prepared through thin-film hydration, which, in addition to the challenges described previously, entails a risk of wasting an excessive amount of material by having to use rotary evaporation, with an impact on costs, especially when postinsertion also includes ligands for active targeting. Another reason for minimizing the amount of material is the limited stability of the micelles over time, as can be seen in Figure S5. It is therefore good practice to freshly prepare them.

The strategy we explored was direct solubilization of DSPE-PEG2000 in PBS, which, unlike thin-film hydration and microfluidics (data not shown), did not require purification of the micelles from organic solvents. This avoided destabilization, material loss, or the preparation of an excessive amount. DLS analysis confirmed the success of the micelle preparation in PBS and was consistent with the literature^39,40^ (Table S7). Interestingly, given the advantages of this preparation method, it could potentially be extended to PEG-lipids functionalized with targeting moieties, and the same applies to the postinsertion method described below. The postinsertion conditions were established to maintain temperature and time compatible with both the stability of the lipids and that of potential drugs that can be encapsulated. Therefore, the temperature was set to 37 °C with an incubation time of 5 h. PEG-lipid insertion efficiency was monitored at 1, 2, 3, 4, and 5 h by analyzing in real-time the reaction mixture through AF4-MD. This allowed to separate PEG-coated liposomes from unincorporated PEG-lipid micelles and to analyze liposome’s size distribution and composition. The coating efficiency was found to increase proportionally with time, reaching a value of over 72% (Figure 5A), while the size of the nanoparticles remained unchanged (Figure 5B).

**Figure 5:**
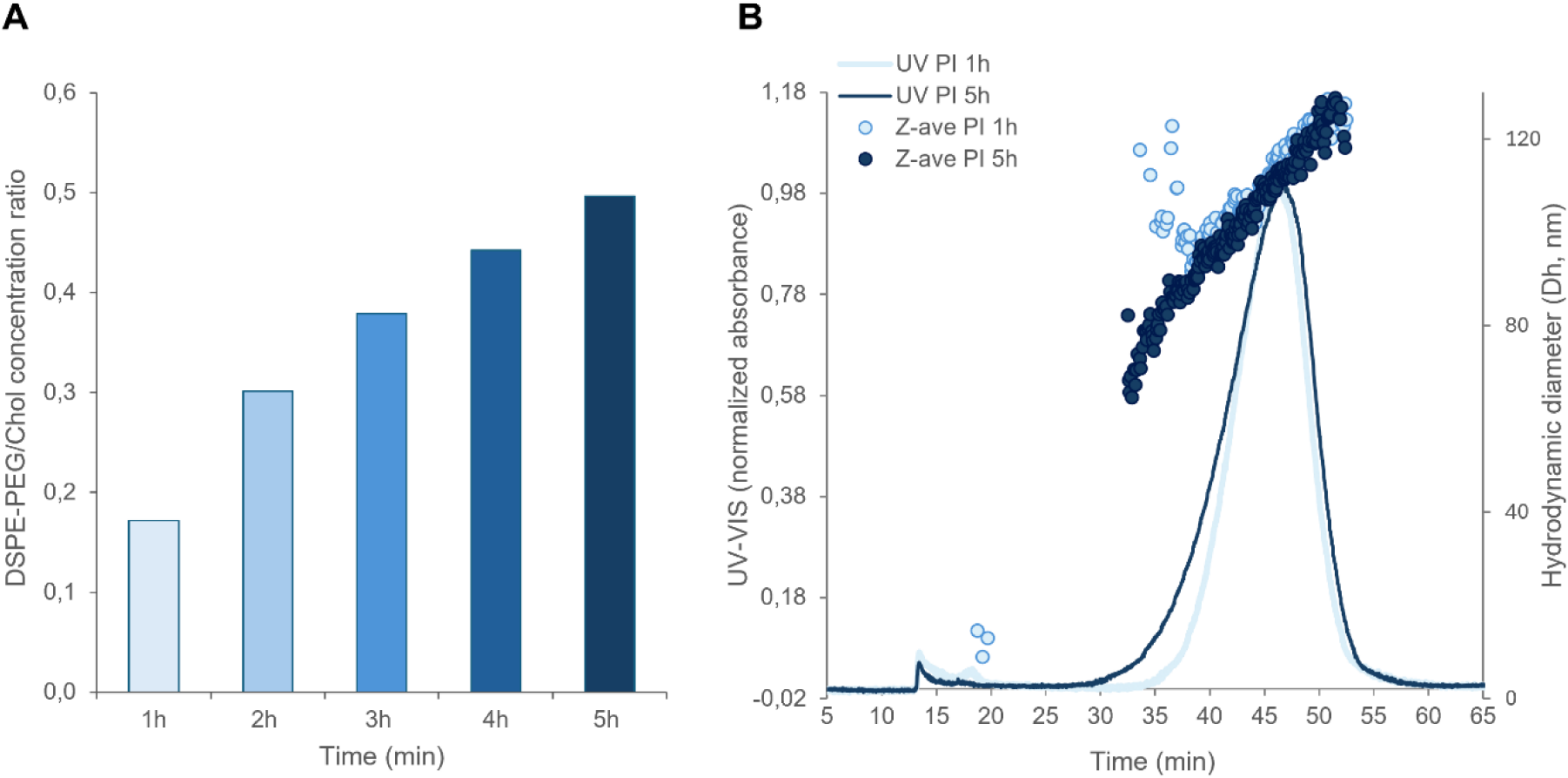
DSPE-PEG2000 postinsertion at a dose of 3 mol % overtime monitoring; A) DSPE-PEG2000/cholesterol molar concentration ratio determined in the liposome fractions by HPLC-CAD after AF4 separation from unincorporated micelles at various incubation times. The increment reflects the increased concentration of postsynthetically inserted PEG-lipid in the formulation; B) AF4-UV-DLS fractograms after 1 and 5 h and particle size of the eluting particle fractions. The line plot represents the UV–vis signal at 280 nm that is in correlation with the concentration of particles, whereas the scatter plot shows the Z-average size of the eluting particles. The elution of smaller than 20 nm micelles can be noted at about 18 min in the 1 h incubation sample, while no micelles are present after 5 h incubation time. The size of the main particle population eluting at about 45 min is very similar (about 110 nm).

Furthermore, at the initial postinsertion time point, when a significant portion of PEG-lipid micelles had not yet been incorporated, the in-line UV and DLS detectors successfully identified their presence (Figure 5B). Therefore, the method’s capability extends beyond separating micelles, as demonstrated in Figure 2 with a spike of concentrated micelles. This spike was intentionally done to facilitate detection and to assess the separation ability of the AF4 setup. In addition, the method can detect micelles at lower concentrations that correspond to the doses used during the formulation phase. Thus, this method is valuable as a preliminary check for potential defects in the postinsertion process (Supporting Information, “Postinsertion tuning”).

It should be noted that with the proportional increase in PEG-lipid incorporation over time, the UV signal also showed a gradual increase. Therefore, we compared the area under the curve (AUC, integrated peak area) of liposomes’ AF4 fractogram peak subjected to postinsertion at 1 and 5 h. The difference was particularly evident with values of 0.32 (0.03) and 0.51 (0.01), respectively (Table S8). We hypothesized that this is due to the increase in coating density, which could affect the way light interacts with the liposomal nanoparticles. In addition, we analyzed DSPE-PEG2000 UV–vis spectra to determine whether the modified lipid could produce a signal in both unimer coil and micelle forms (Figure S6) and found that DSPE-PEG2000 shows absorption at this wavelength even in the dissolved form.

We can therefore conclude that among the in-line detectors, UV can provide additional supportive information for a preliminary assessment of postinsertion outcome. Specifically, the disappearance of the UV peak corresponding to unincorporated PEG micelles, along with an increase in the AUC of the peak corresponding to the pegylated liposomes, can indicate potentially effective postinsertion, which is subsequently confirmed though HPLC-CAD.

Having established 5 h as the stop incubation time, postinsertion was carried out with two commonly used doses, namely 3 mol % and 6 mol % PEG. At the selected doses, PEG chains likely adopt the “mushroom” configuration and represent the right compromise between imparting stealth properties to the liposome and not limiting cellular uptake and less efficient binding with protein targets, which can be caused by excessive PEGylation.^41^ Postinsertion efficiency was over 75% in both cases, demonstrating that the stop incubation time was also suitable for a double dose (Figure S7). Also in this case, the AUC was comparable between doses and higher than that of the liposome before postinsertion, the size remained unchanged, and the zeta potential shifted toward more negative values (Table 1 and Figure S8).

**Table 1:** Characterization of Liposomes (*n* = 3) Postinserted with 3% and 6 mol % PEG, Presenting Critical Quality Attributes (CQAs) Such As Postinsertion Efficiency, Hydrodynamic Size Determined via DLS Batch Mode, and Zeta Potential, with Standard Deviations Indicated in Brackets^a^

	Postinsertion efficiency (%)	Size (nm), PDI	Zeta potential (mV)	AUC (V·min)
0.2 mol % PEG	-	102 (0.5), 0.053 (0.01)	–9 (1.8)	0.4 (0.05)
3 mol % PEG	76 (4)	105 (11), 0.072 (0.02)	–22 (0.1)	0.5 (0.01)
6 mol % PEG	91 (4)	99 (6.4), 0.075 (0.02)	–22 (0.5)	0.5 (0.07)

a The area under the curve (AUC) is included in the Supporting Information for the characterization.

Additionally, two low doses, 0.75 and 1.5 mol %, and a higher dose, 10 mol % PEG, were tested (Figure S9). Unlike the low doses, where PEG-lipid incorporation was complete, at 10 mol % the efficiency decreased significantly, and the purification capacity of centrifugal ultrafiltration was also limited. This is probably due to surface saturation, as explored in depth using advanced characterization techniques like ToF-SIMS.^13^ In fact, at excessive PEG doses, the liposomes reach an optimal configuration, with PEG chains passing from a “mushroom” to a “brush” configuration. This change results in a denser and fuller surface coverage, preventing the incorporation of additional PEG-lipids.

To demonstrate the versatility of the approach, DSPE-PEG5000, which like DSPE-PEG2000 is commonly used in liposomal formulations, was investigated following the same workflow, starting with the preparation of DSPE-PEG5000 micelles in PBS and incubation at 37 °C at a dose of 3 mol %. After 5 h of incubation, the postinsertion efficiency was just under 50%, so the incubation time was extended to 7 h reaching complete efficiency (99%). As already observed for DSPE-PEG2000, the AUC increased with increasing PEG-lipid incorporation, with values of 0.47 and 0.61 after 5 and 7 h, respectively. The size distribution obtained by in-line DLS analysis remained the same.

The experimental setup described here could be well suited to a laboratory workflow enabling real-time monitoring of the PEG-lipid postinsertion and liposome integrity to determine whether postinsertion adversely impacted nanoparticle stability. Although fine-tuning of the postinsertion process to specific PEG coating densities and liposome compositions^10^ was not the focus of this work, our goal was to provide a reference approach that can be used as a starting point for adapting to other lipid-based formulations compatible with AF4 and HPLC-CAD. It is worth noting that HPLC-CAD-based analysis is suitable for a wide range of lipids^34^ commonly used in both liposomes and solid lipid nanoparticles.

The chromatographic separation of lipid components requires careful optimization, particularly when formulations contain the same lipids conjugated to PEG chains of varying lengths, which may coelute. In this work, we propose a gradient elution method capable of separating DSPE-PEG2000 from DSPE-PEG5000, which can help optimize the elution protocols for other systems.

In conclusion, the offline integration of HPLC-CAD with AF4 expands analytical possibilities for lipid-based drug delivery systems, making this workflow highly versatile. Moreover, this strategy only requires a minimal amount of material, since small aliquots from the same incubation mixture can be injected directly into AF4, after a compatibility check with the mobile phase, without requiring any sample preparation step that could potentially alter it. This aspect may be interesting in terms of both time and cost savings if ligands such as antibodies need to be added during postinsertion.^42^

### Validation of Dialysis and Centrifugal Ultrafiltration

Unlike industrial production, where continuous purification is the strategy of choice, dialysis and centrifugal ultrafiltration are traditional purification methods commonly used on a laboratory scale in nanoparticle formulation workflows.^43^ They are gentle, compatible with sterile conditions, and do not disrupt nanoparticles, provided that suitable membrane and filter materials are selected.

In the formulation strategy developed here, we used both methods for different purposes. Dialysis was used to purify the liposome containing 0.2 mol % PEG synthesized through microfluidics in order to remove the ethanol used in the synthesis and any unincorporated lipid components. Centrifugal ultrafiltration, on the other hand, was employed to purify PEGylated liposomes after postinsertion from any residual PEG-lipids. The latter method is particularly relevant because it allows the nanoparticles to be concentrated after washing, even under sterile conditions, unlike that for AF4, which causes excessive dilution. For this reason, AF4 will be used solely for analytical purposes within the workflow to validate these purification methods, given the importance of complete nanoparticle purification to avoid any bias in quality control.

AF4 was selected for this purpose because the continuous flow of the mobile phase prevents the risk of saturation and adsorption of materials on the membrane/filter, which can occur in the traditional purification methods. In addition, the ability of AF4 to separate PEGylated liposomes from unincorporated PEG-lipids based on size has already been demonstrated (Figures 2 and 5B).

To evaluate the efficiency of the dialysis, we compared the lipid composition of the 0.2 mol % PEG liposome subjected to 24 h dialysis with that purified through AF4, following the liquid–liquid extraction protocol (Figure 6A).

**Figure 6:**
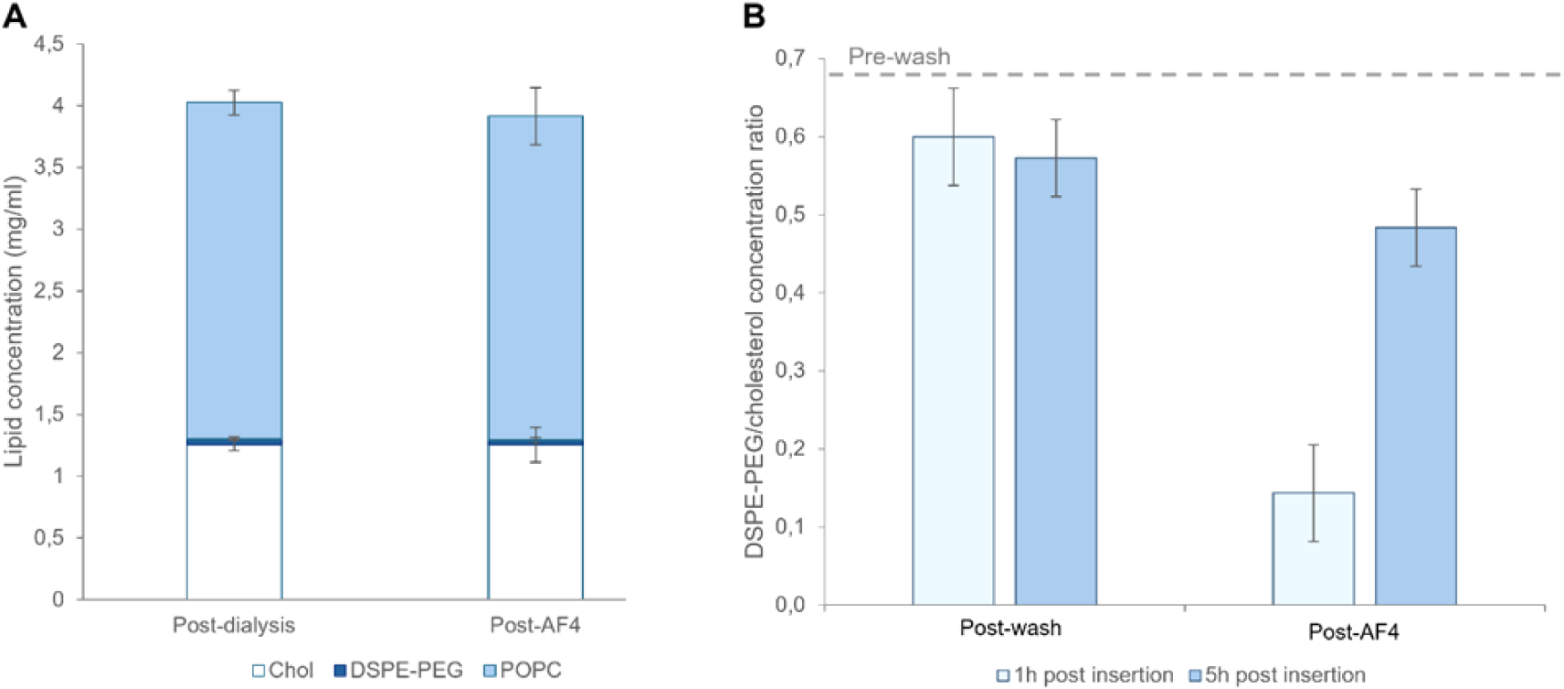
A) Total lipid concentration of liposome batch 0.2 mol % PEG postdialysis and post-AF4; The various colors represent the contribution of the single components (white—cholesterol, dark blue—DSPE-PEG, light blue—POPC). No composition difference is observed between samples purified by dialysis and AF4 separation; B) Comparison of efficient and inefficient postinsertion: DSPE-PEG2000/cholesterol ratio determined by HPLC-CAD in liposome batches with 3 mol % PEG-lipid in postinserted samples after 1 h (light blue) and 5 h (dark blue) incubation times, before (dashed line) and after centrifugal ultrafiltration (bars on the left) or AF4 separation (bars on the right). Centrifugal ultrafiltration was not able to remove the PEG-lipid micelles, thus the time dependence of the process is not traceable. AF4 purification separates the liposomes from micelles, allowing the observation of the PEG-lipid insertion in time (difference between light and darker blue bars).

Since both the total lipid concentration and the lipid/cholesterol ratio remained unchanged, with no loss of lipids (POPC, cholesterol, or PEG-lipids), we were able to conclude that the dialysis effectively purified the liposomes. Furthermore, it was possible to assess that the synthesis efficiency was complete, confirming that, despite the deviation from the typical liposomal composition with 3 mol % PEG, the reduction of the content down to 0.2 mol % had no negative effect on the synthesis.

To validate the centrifugal ultrafiltration approach, we compared the lipid composition of liposomes subjected to a 5 h postinsertion followed by centrifugal ultrafiltration with those purified through AF4. The lipid ratio remained constant, thus demonstrating that the ultrafiltration process did not adversely affect the integrity of the nanoparticles, ensuring reliable washing and purification outcomes (Table 2).

**Table 2:** Lipid Ratio of Liposome Batch Subjected to a 5 h Postinsertion with 3 mol % DSPE-PEG2000 Postcentrifugal Ultrafiltration and Post-AF4

Lipid/cholesterol ratio	Post-AF4	Postwash
Mean DPSE-PEG2000 (SD)	0.50 (0.07)	0.52 (0.10)
Mean POPC (SD)	2.13 (0.04)	2.14 (0.23)

To study centrifugal ultrafiltration in greater depth, its efficiency was also evaluated in an intentionally defective postinsertion scenario, characterized by a higher concentration of unincorporated DSPE-PEG2000 in the incubation mixture requiring purification.

As the monitoring of the coating over time (Figure 5A) showed that after 1 h only 25% of PEG-lipid was inserted in the surface of the liposomes, this time point was selected to simulate the aforementioned, inefficient postinsertion and put into comparison with an efficient 5 h postinsertion (Figure 6B).

Purification by centrifugal ultrafiltration proved reliable only after 5 h incubation, suggesting that the presence of residual DSPE-PEG micelles hinders the washing.

We assume that if the micelles have integrated only minimally into the liposome phospholipid bilayer and considering their mean hydrodynamic radius of 14 nm, they may not be able to pass through the pores of the 100 kDa regenerated cellulose filter used. If, on the other hand, the insertion of PEG-lipids was effective, as in the case of 5-h incubation, probably due to the strong dilution that occurs during centrifugal ultrafiltration washing, the PEG-lipids are effectively removed because they are present in single-coil unimer form.^39^

To conclude, AF4 proved valuable for analytical purposes, as it fulfilled a dual role by assessing PEG-lipid insertion efficiency and verifying the purification quality achieved through dialysis and centrifugal ultrafiltration washing. In conclusion, during the optimization of the postinsertion method, adjusting PEG dose and incubation times can enhance coating efficiency and establish reliable washing conditions, which in turn facilitates subsequent reliable physicochemical characterization.

### Stability in Physiologically Relevant Media

Unlike batch-mode DLS, AF4-MD is known to be a technique particularly suitable for assessing the physical stability, in terms of size distribution and drug release, of nanopharmaceuticals in complex biological media, whose integrity can be severely compromised by serum proteins.^23^ Measurement resolution is greatly improved compared to batch-mode DLS, as AF4 separates free plasma proteins from nanoparticles prior to the size measurement analysis.

We therefore decided to explore this analytical application by extending it to chemical composition analysis to verify that the DSPE-PEG2000 incorporated via postinsertion was stably integrated into the phospholipid bilayer following incubation in cell culture medium containing bovine serum at 37 °C.

Through in-line UV detection, we monitored the separation of serum proteins, which are potentially complexed with liposomes,^44^ from free excess proteins. Liposome components were extracted from the serum protein suspension through liquid–liquid extraction of lipids to prevent the interference of precipitating proteins with HPLC-CAD analysis. Both size distribution and chemical analysis showed that the stability and composition of the liposomes, including PEG-lipids, remained unchanged (Figure 7).

**Figure 7:**
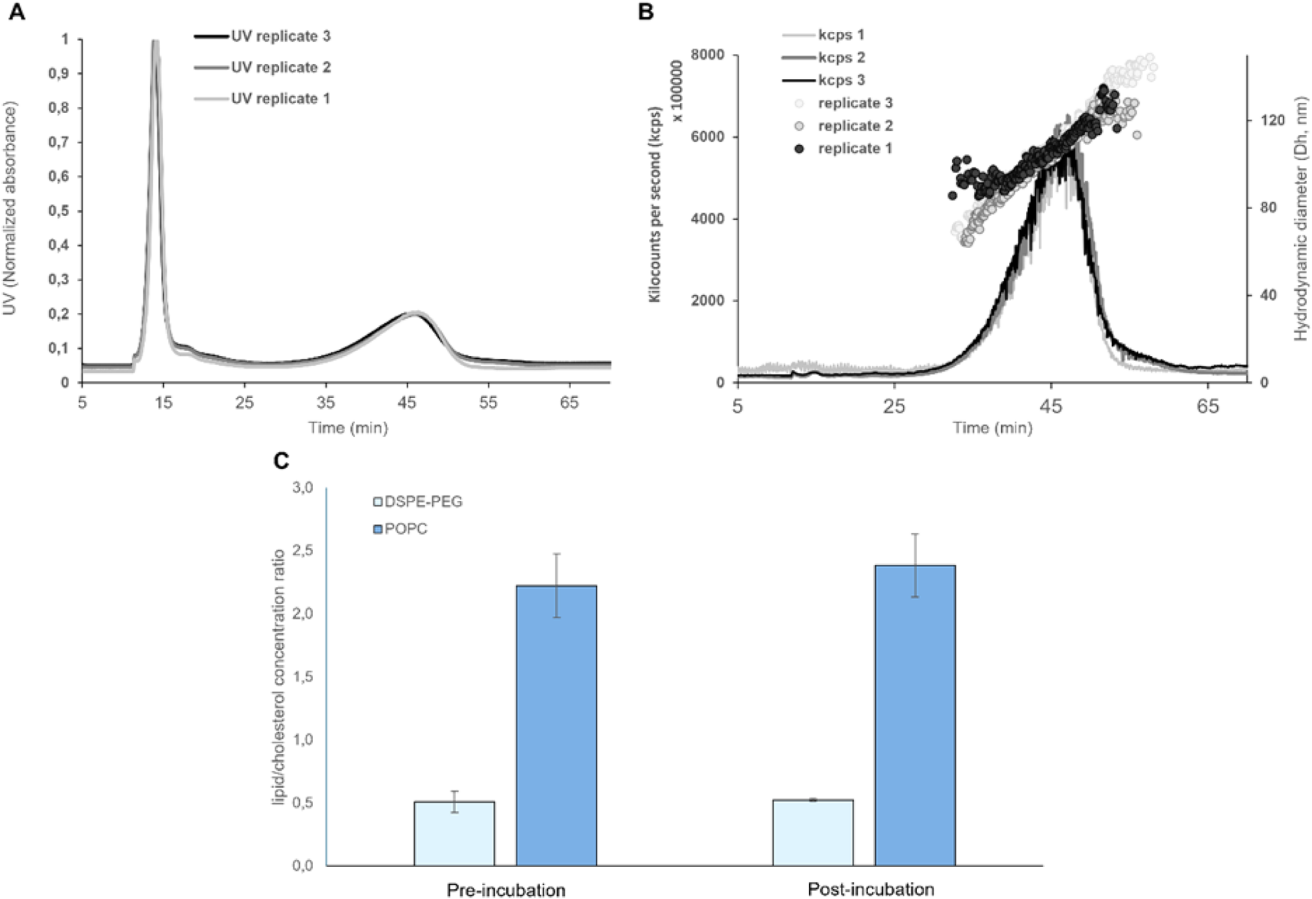
Liposome batch 3 mol % PEG stability before and after overnight incubation in cell culture media. A) AF4-UV of 3 replicates after incubation showing unbound serum proteins and liposomes eluting at 15 and 46 min, respectively; B) In-line DLS light scattering intensity of the incubated liposomes and the scatter plot representing the Zeta-average; C) Lipid composition (lipid/cholesterol ratio).

### Morphology Characterization

Liposomes are metastable nanostructured systems that depend on both the hydrophobic/hydrophilic balance of the lipid components and the synthesis strategy selected, as described in the literature.^45^

In our formulation protocol, we started with a typical liposomal composition and then pushed it to the limit by minimizing the amount of PEG-lipid, and we then verified its compatibility with microfluidic synthesis. Furthermore, this composition had an impact on the structural and dynamic transitions of the liposomal phospholipid bilayer^45^ that occurred during postinsertion conducted at intentionally mild temperatures.

It was therefore crucial to assess whether these changes to the composition and the postinsertion process did not compromise the morphology of the liposomes. The liposomes, as seen in Figure 8, maintain a typical unilamellar spherical hollow structure with a size of approximately 100 nm, in accordance with DLS analysis in both batch and in-line modes.

**Figure 8:**
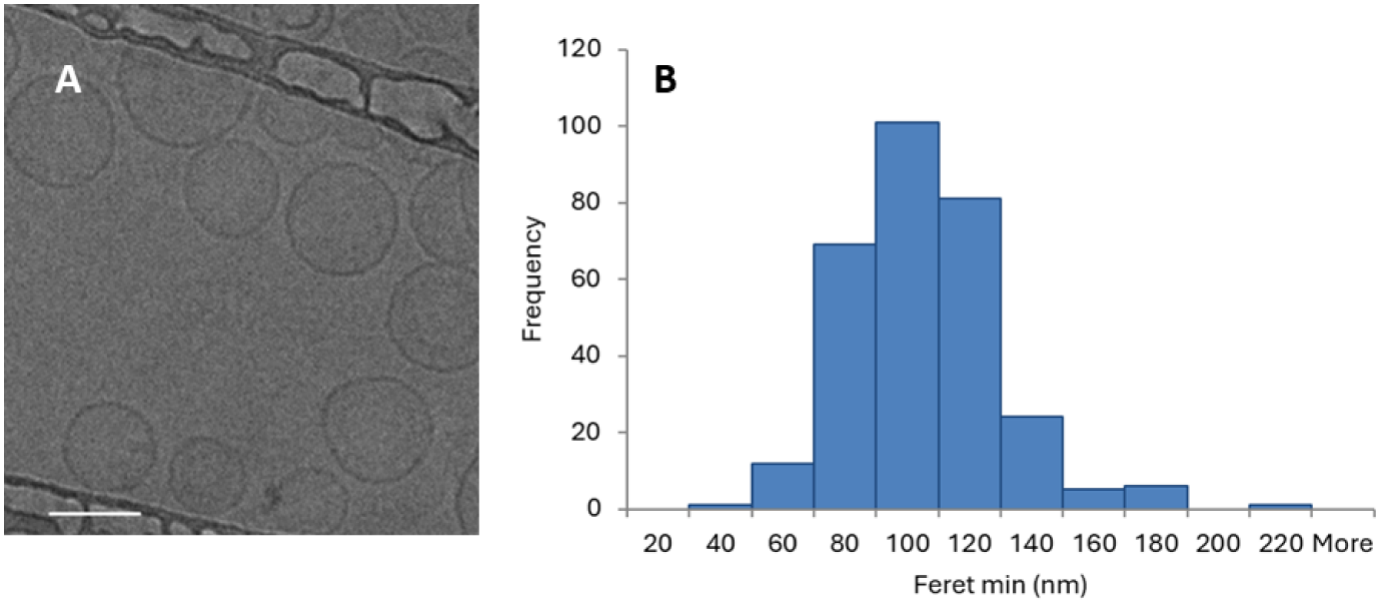
Cryo-TEM picture of postinserted liposomes, scale bar 100 nm. Mean Feret min (94.8 ± 24.3 nm).

## Conclusions

The composition of nanocarriers is as important as the drug they carry since the therapeutic effect and safety derive from the synergistic interaction of both. Therefore, inadequate characterization of these components can present a major obstacle to nanomedicine formulation development. With this understanding, the scientific community is actively seeking analytical solutions that go beyond the basic analysis of average nanoparticle composition to ensure adequate quality control. By integrating separative techniques with combined detectors, researchers aim to gather the most detailed information possible at the nanoparticle level on critical quality attributes. This study points in this direction, with particular attention paid to exposed components, such as the PEG-lipid coatings, a CQA of key importance according to EMA’s reflection papers.

To demonstrate the full potential of our analytical workflow in a straightforward way, we adopted a strategic approach to liposome synthesis. By opting for postinsertion, we minimized potential biases associated with preinsertion methods, such as an uneven PEG chain orientation. Additionally, we verified the compatibility of microfluidic synthesis, a widely used strategy, with the postinsertion coating process, ensuring a reliable and efficient method for producing PEGylated liposomes.

AF4-MD is central to our analytical method, given its growing importance in both research and regulatory fields, and it takes into account the existing SOPs. It should be noted that the liquid–liquid extraction proposed here allows for the expansion of the analytical cascade reported in the updated ISO standard (ISO 21362:2026) concerning offline analyses of AF4 fractions, enabling in-depth analysis of liposomal composition post size-based fractionation via HPLC-CAD. The added value of our workflow lies in its application within the pharmaceutical field, specifically in verifying PEG-lipid incorporation for quality control of coatings, which could potentially be adapted to other lipid-based nanoparticles.

This combined AF4-MD/HPLC-CAD workflow presents a promising solution for nanomedicine developers, providing a practical platform for routine quality control that combines size and composition analyses into a single solution. This information improves the current best-practice toolbox, which includes batch-mode DLS, indirect PEG assays, and separate purification assessments.

In detail, in the context of postinsertion tuning, by effectively separating PEG-lipid micelles from liposomes using AF4, our method enables precise quantification of the postinserted PEG-lipid fraction through HPLC-CAD analysis. Additionally, it allows for monitoring of residual micelles in cases of unsuccessful postinsertion and confirms the preservation of size and structural integrity. This feedback facilitates data-driven adjustments of key postinsertion variables such as PEG-lipid dose, incubation temperature and time, and purification techniques. Moreover, the HPLC-CAD analysis of AF4-collected fractions provides absolute lipid-to-PEG-lipid molar ratios, enabling developers to verify that critical quality attributes meet specifications prior to scale-up.

Furthermore, our experimental setup is designed to evaluate liposomes both in their original buffer and within stability-testing environments, thereby facilitating the prediction of efficacy and safety, as required by the pharmaceutical sector. Consequently, our workflow holds the potential to shorten the formulation process, reduce material waste, and generate a robust, regulatory-compatible data set that surpasses the qualitative, bulk-averaged assessments typical of existing practices.

## Supplementary Material



## Data Availability

The data supporting this study are available in https://data.jrc.ec.europa.eu/dataset/a0ec9618-e564-4baa-9811-2737cedeff0a.

## Notes

Additional experimental details on liposome characterization, postinsertion, liquid–liquid extraction and HPLC-CAD and AF4 methods (PDF)

## Abbreviations

AF4: Asymmetric-flow field-flow fractionation
MD: multidetector
PEG: polyethylene glycol
DLS: Dynamic Light Scattering
HPLC-CAD: High-Performance Liquid Chromatography-Charged Aerosol Detector
ELS: Electrophoretic Light Scattering
RSD: relative standard deviation
AUC: area under the curve
CQA: critical quality attributes
