# Supplementary material for: In-Depth Characterization of PEGylated Liposomes: Using AF4 and HPLC-CAD in Tandem as a Strategy for Composition Assessment and Postinsertion Optimization
Source: Anal Chem. 2026 Jun 25;98(26):19427–39. doi: 10.1021/acs.analchem.5c07710 (PMC13347696; doi:10.1021/acs.analchem.5c07710)
Supplement: Supplementary file 1 [file ac5c07710_si_001.pdf]

SUPPORTING INFORMATION

In-Depth Characterization of PEGylated Liposomes: Using AF4 and HPLC-CAD in Tandem as a Strategy for Composition Assessment and Post-Insertion Optimization

Silvia L. Appleton[1], Guillaume Bucher[1], Jessica Ponti[1], Dora Mehn[1]*

[1]European Commission, Joint Research Centre (JRC), 21027 Ispra, Italy.
silvia.appleton@ec.europa.eu; guillaume.bucher@ec.europa.eu; jessica.ponti@ec.europa.eu;
*dora.mehn@ec.europa.eu

Table of Contents

Synthesis of liposomes with low-PEG content via microfluidics S2
Development of AF4-MD method S2
Optimization of lipid extraction from AF4 fractions and HPLC-CAD analysis S4
Post-insertion tuning S8

Synthesis of liposomes with low-PEG content via microfluidics

Table S1. Mean and standard deviation of size, polydispersity index (PdI) and zeta potential of liposomes with different PEG densities.

Fomulation code	*Batch mode DLS (Dh in nm)*		*ELS (mV)*
	Z-average (SD)	PdI (SD)	Zeta potential (SD)
Liposome 0 mol% PEG	121 (1)	0.225 (0.011)	-4 (0.5)
Liposome 0.2 mol% PEG	90 (9)	0.059 (0.014)	-9 (1.8)
Liposome 3 mol% PEG	93 (10)	0.062 (0.022)	-18 (0)

Development of AF4-MD method

Table S2. AF4 separation experiment with and without crossflow for recovery analysis, repeatability assessment for retention time and size using a liposome batch 0.2 mol% PEG.

	Replicate 1	*Replicate 2*	*Replicate 3*	*Mean*	*SD*
Area under the curve w/o crossflow	0.54	0.54	0.53	0.53	0.01
Area under the curve	0.32	0.39	0.44	0.38	0.06
Retention time (min)	43	43	43	43	0.08
Hydrodynamic diameter	103	102	102	103	0.5

Table S3. Hydrodynamic size comparison between batch mode and in line DLS liposome batch 0.2 mol% PEG.

Formulation code	*Batch mode DLS (Dh in nm)*		*AF4-DLS (Dh in nm)*	
Liposome 0.2 mol% PEG	Cumulant analysis		Intensity based PSD	
	Z-average (SD)	PdI (SD)	Mean (SD)*	D_{min}, D_{max}*
	102 (0.5)	0.053 (0.014)	103 (1.4)	91, 118

*Calculated by averaging the diameter over the Full Width at Half Maximum (FWHM) of the liposome elution peak over 3 replicates.

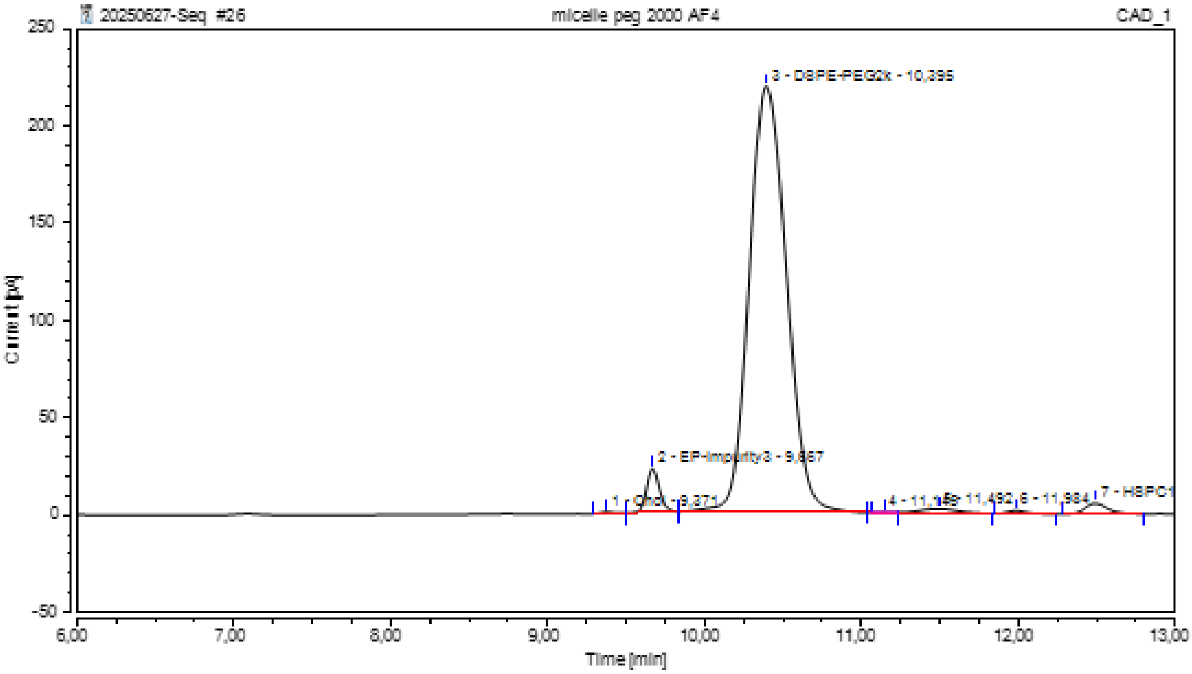

Figure S1. HPLC-CAD analysis of the AF4 fraction eluting at 18.5 minutes, confirming DPSE-PEG2000 peak identity.

Optimization of lipid extraction from AF4 fractions and HPLC-CAD analysis

Phase A	Acetonitrile/Water 9/1 + 5 mM Ammonium Acetate
Phase B	Methanol + 5 mM Ammonium Acetate
Flowrate	0.7 mL/min

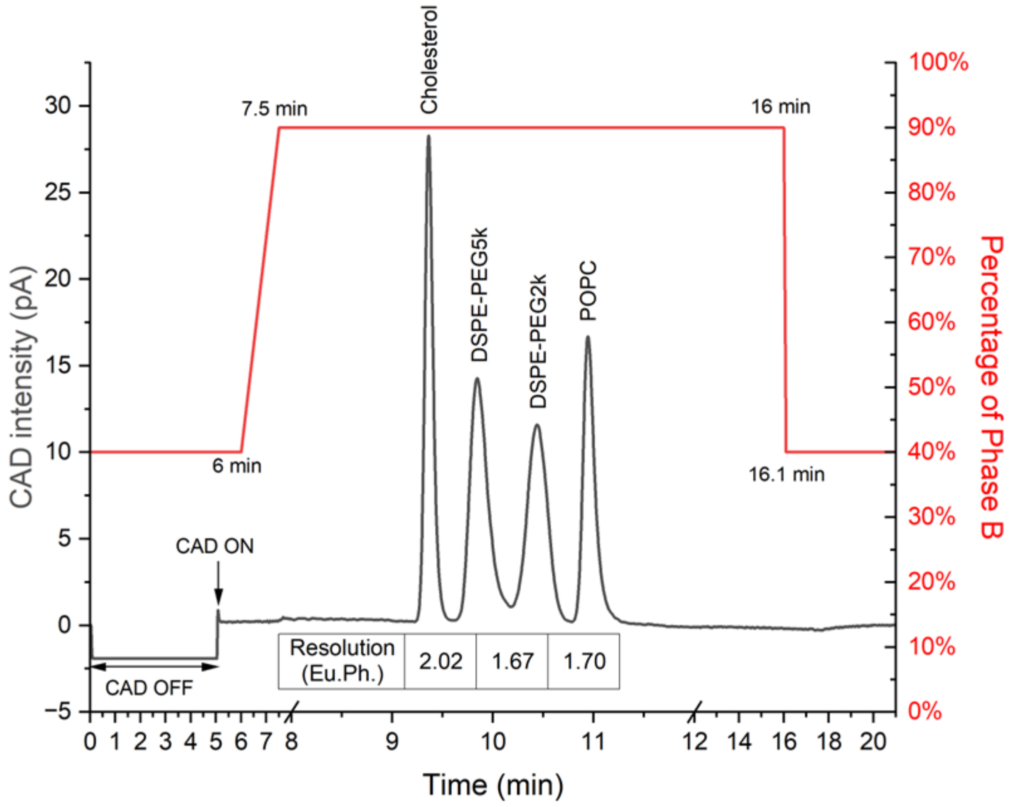

Figure S2. HPLC-CAD chromatograms of liposome lipid components after dissolution in methanol for composition analysis.

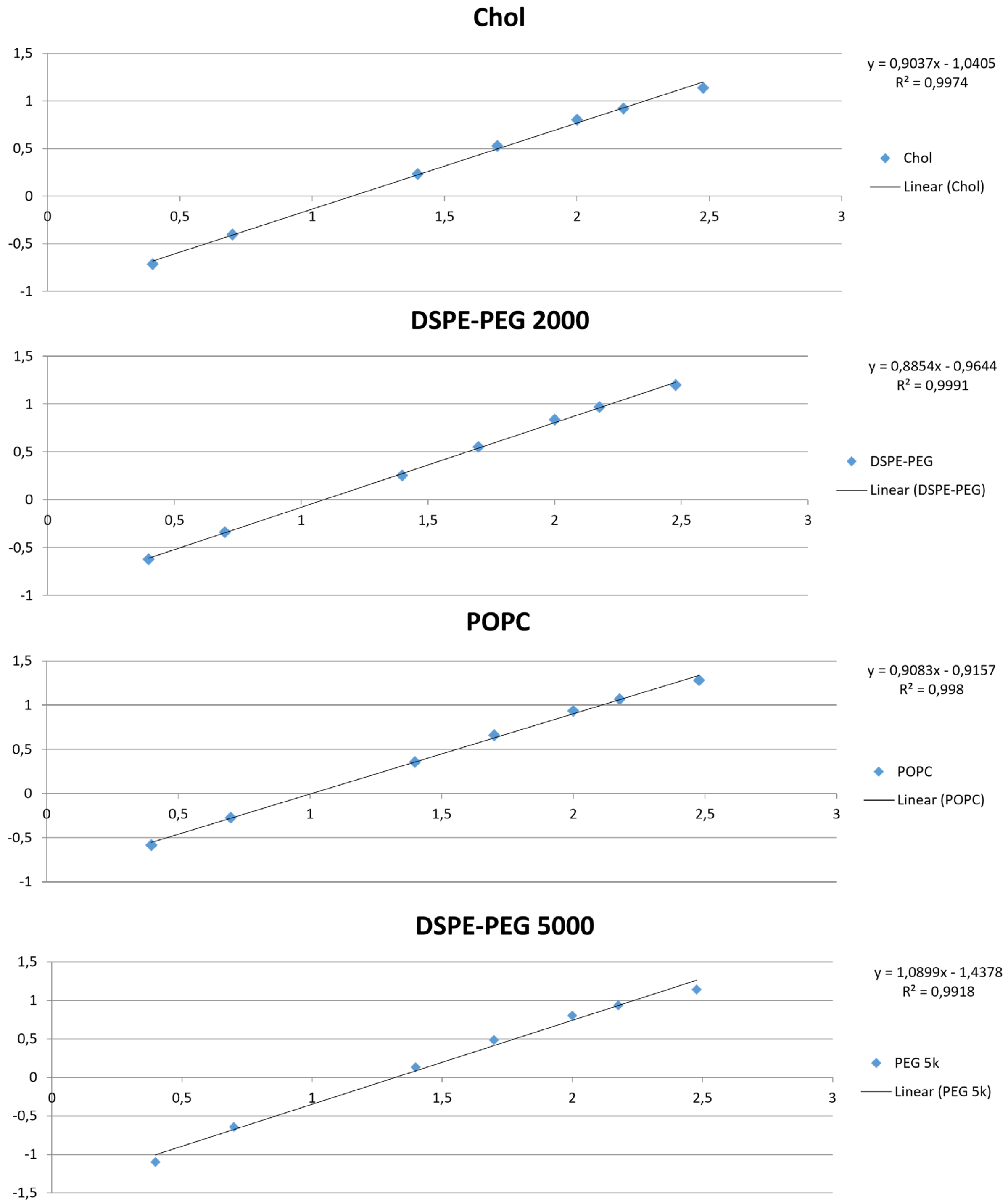

Figure S3. HPLC-CAD calibration curves of standard lipid mixtures.

Table S4. Absolute recovery after Bligh and Dyer extraction of cholesterol, DSPE-PEG2000 and POPC.

Abs. Recovery	D200	D80	D40	D20
**Cholesterol**	73%	74%	91%	78%
**DSPE-PEG**	41%	57%	81%	78%
**POPC**	87%	79%	93%	78%

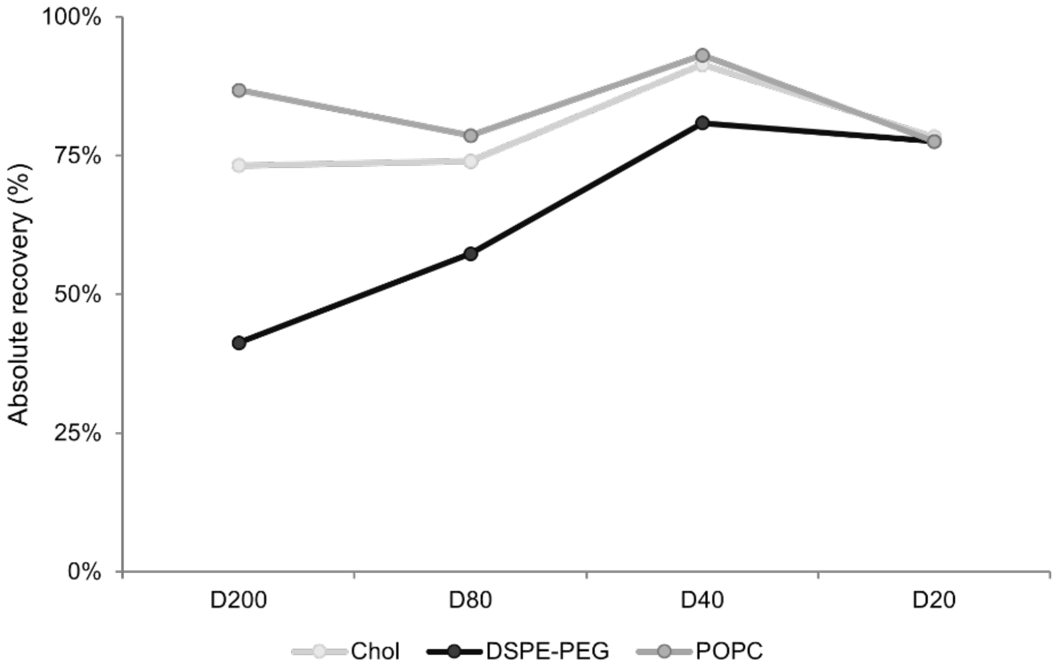

Figure S4. Absolute recovery of cholesterol, DSPE-PEG2000 and POPC extracted from 3 mol% PEG liposomes diluted 1:20, 1:40, 1:80 and 1:200 in PBS using Bligh and Dyer method.

Table S5. Absolute recovery after liquid-liquid extraction of cholesterol, DSPE-PEG2000 and POPC.

Abs. Recovery	D1000	D400	D200	D100	Average	SD	%RSD
**Cholesterol R1**	96%	91%	95%	92%			
**Cholesterol R2**	92%	81%	88%	88%	89%	5%	5%
**Cholesterol R3**	93%	84%	87%	82%			
Abs. Recovery	D1000	D400	D200	D100	Average	SD	%RSD
**DSPE-PEG R1**	87%	89%	95%	95%			
**DSPE-PEG R2**	83%	77%	87%	90%	87%	6%	7%
**DSPE-PEG R3**	88%	77%	86%	88%			
Abs. Recovery	D1000	D400	D200	D100	Average	SD	%RSD
**POPC R1**	71%	82%	88%	84%			
**POPC R2**	85%	74%	80%	80%	79%	5%	6%
**POPC R3**	77%	74%	79%	77%			

Table S6. Relative recovery normalized to cholesterol content after liquid-liquid extraction of DSPE-PEG2000 and POPC.

Rel. Recovery	D1000	D400	D200	D100	Average	SD	%RSD
**DSPE-PEG R1**	90%	98%	100%	103%			
**DSPE-PEG R2**	91%	95%	99%	102%	98%	5%	5%
**DSPE-PEG R3**	95%	93%	99%	106%			
Rel. Recovery	D1000	D400	D200	D100	Average	SD	%RSD
**POPC R1**	74%	90%	92%	91%			
**POPC R2**	93%	91%	92%	91%	89%	5%	6%
**POPC R3**	83%	89%	91%	94%			

Post-insertion tuning

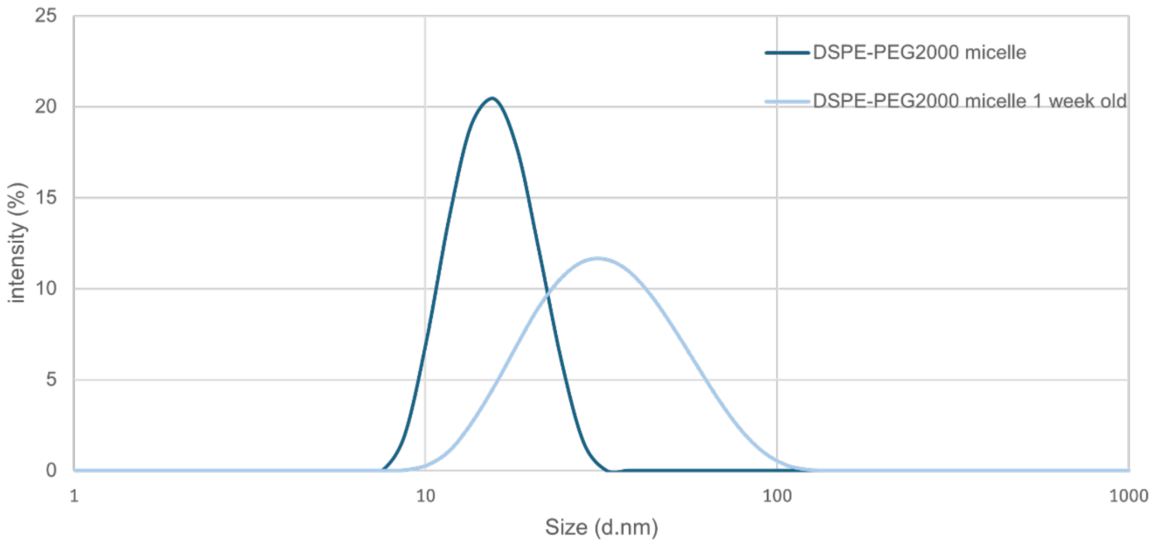

Figure S5. Intensity based particle size distribution by batch mode DLS of DSPE-PEG2000 micelle synthesized through direct solubilization in PBS and its instability after 1 week of storage at 4°C.

Table S7. Mean hydrodynamic size and polydispersity index of DSPE-PEG2000 micelle in PBS.

	Batch mode DLS (Dh in nm)	
DSPE-PEG2000 micelle	Cumulant analysis	
	Z-average (SD)	PdI (SD)
	14 (0.2)	0.04 (0.03)

Table S8. Area under the curve of liposomes at t0, and those subjected to post-insertion at 1 and 5 hours, measured using the UV detector at 280 nm in line with AF4.

PI time	**0h**	**1h**	**5h**
	0.37	0.3	0.5
	0.37	0.31	0.52
	0.31	0.34	0.52
mean AUC	0.35	0.32	0.51
SD	0.03	0.02	0.01

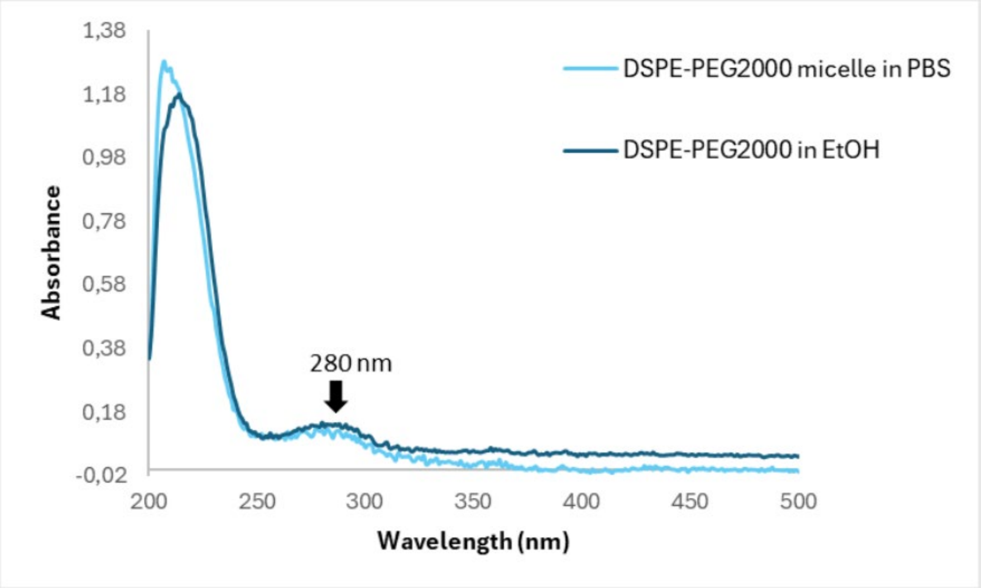

Figure S6. DSPE-PEG 2000 UV-VIS fractogram in ethanol and PBS at a concentration of 28.3 mg/ml.

As far as the limit of detection (LOD) of micelles is concerned, it was estimated under the experimental condition in which the micelles were detectable through the combination of UV

and light scattering signals, that is, after 1 hour of post-insertion (as shown in Figure 5B). This amount can be calculated knowing the PEG-lipid concentration already present in the liposomes (0.2 mol%), the amount of PEG-lipid added in the form of micelles (3 mol%), the total amount of PEG-lipid successfully post-inserted following 1 hour and 2 hours of incubation, the AF4 sample dilution factors, and the volume of injection for AF4 analysis (20 µL). As a result, we were able to estimate through HPLC-CAD the absolute LOD for PEG-lipid micelles in AF4-UV-DLS to range between 4.2 and 6.1 µg PEG-lipid. This LOD range was confirmed by injecting a known amount of PEG-lipid micelles (141.7 µg) in the AF4 system (as in Figure 2), comparing the peak retention time and using the peak area to determine the amount of PEG-lipid micelles eluting after 1 hour of post-insertion (AUC_a=0.062, AUC_b=0.003). This amount was estimated to be 6.8 µg PEG-lipid, which is in line with the value obtained from HPLC-CAD measurements (i.e. 6.1 µg).

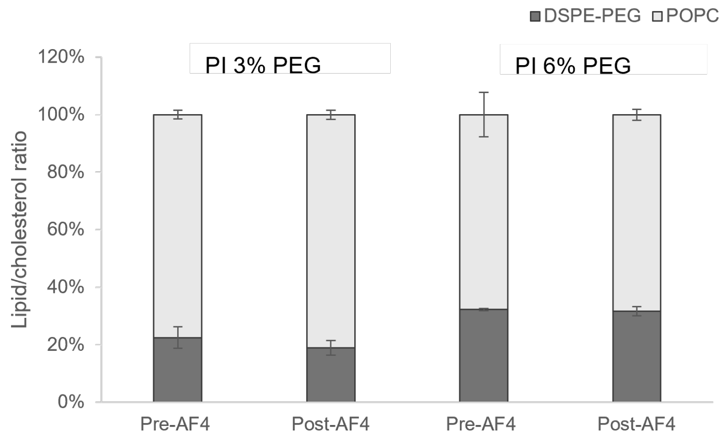

Figure S7. Composition of liposomes post-inserted with 3 mol% and 6 mol% DSPE-PEG2000 determined in the liposome fractions by HPLC-CAD after AF4 separation.

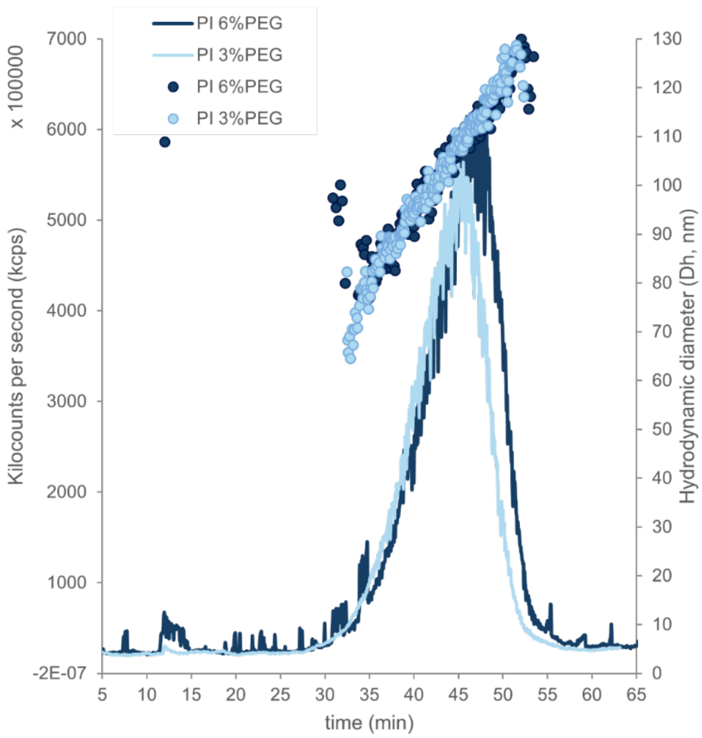

Figure S8. AF4-DLS fractograms of liposomes post-inserted with 3 mol% and 6 mol% DSPE-PEG2000, the line plot represents the UV-Vis signal at 280 nm that is in correlation with the concentration of particles, whereas the scatter plot shows the Z-average size of the eluting particles.

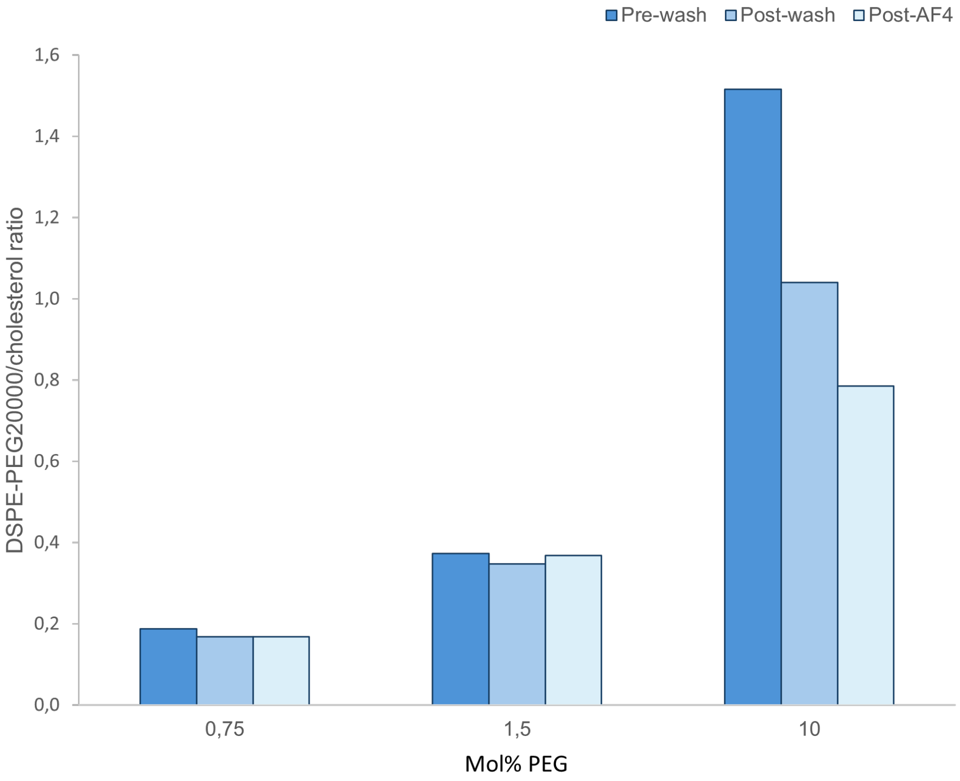

Figure S9. PEG-lipid/cholesterol ratio of liposomes post-inserted with 0.75, 1.5 and 10 mol% DSPE-PEG2000 before and after washing/AF4.

Table S9. Conditions used for MD-AF4 measurements

Parameters	
Eluent	Phosphate buffer saline pH=7.4
Membrane	10kDa RC
Channel length	275 mm
Spacer	350 µm
Injection flow	0.2 ml/min
Channel Flow	1 ml/min
Focus step	
Injection flow	0.2 ml/min
Injection time	10 min
Cross Flow	1 ml/min
Focus Pump	1.30 ml/min
Transition pump	1 min
Elution step	
Step 1: Linear cross flow (exponent 1)	1mL/min for 0.5 minutes
Step 2: Power cross flow (exponent 0.5)	1mL/min from 0.5 to 60 minutes
Step 3: Cross flow	0 mL/min from 60 to 80 minutes
